# Effect of an anti-methanogenic supplement on enteric methane emission, fermentation, and whole rumen metagenome in sheep

**DOI:** 10.3389/fmicb.2022.1048288

**Published:** 2022-11-21

**Authors:** Pradeep Kumar Malik, Shraddha Trivedi, Atul Purushottam Kolte, Archit Mohapatra, Raghavendra Bhatta, Habibar Rahman

**Affiliations:** ^1^ICAR-National Institute of Animal Nutrition and Physiology, Bangalore, India; ^2^International Livestock Research Institute, New Delhi, India

**Keywords:** anti-methanogenic supplement, methane, metagenome, sheep, tannin

## Abstract

A study was conducted to investigate the impact of an anti-methanogenic product supplementation on enteric methane emissions, whole rumen metagenome and ruminal fermentation in sheep. Twelve adult male sheep were randomly divided into two groups of six animals each. Animals were fed *ad libitum* on a total mixed ration either without (CON) or with an anti-methanogenic supplement (Harit Dhara-HD). The anti-methanogenic supplement contained 22.1% tannic acid in a 3: 1 ratio of condensed and hydrolysable tannins. The supplementation of product revealed a significant reduction in daily enteric methane emission (21.9 vs. 17.2 g/d) and methane yield (23.2 vs. 18.2) without affecting the nutrient intake and digestibility. However, the propionate concentration in the HD treatment group was significantly higher than in the CON group. On the contrary, the ammonia nitrogen concentration was lower. The anti-methanogenic supplement significantly decreased the ruminal protozoa in the HD treatment group. Whole rumen metagenome analysis revealed that the core bacterial (*Bacteroidetes* and *Firmicutes*) and archaeal communities (*Methanobrevibacter* and *Methanosarcina*) were comparable between the CON and HD treatment groups. However, the supplementation of anti-methanogenic product led to a considerable reduction in the abundance of *Proteobacteria*, whereas the abundance of *Lentisphaerae* was greater. The supplementation significantly decreased the abundance of *Methanocaldococcus*, *Methanococcoides*, *Methanocella*, and *Methanoregula* methanogens. A total of 36 KO related to methanogenesis were identified in this study. The activities of formate dehydrogenase (EC 1.8.98.6) and tetrahydromethanopterin S-methyltransferase (EC 2.1.1.86) were significantly lowered by the anti-methanogenic product supplementation in sheep. In conclusion, the anti-methanogenic supplement has the potential to decrease enteric methane emission (~22%) at the recommended level (5% of DM) of supplementation. The contribution of minor methanogens vulnerable to supplementation to rumen methanogenesis is not known; hence, the culturing of these archaea should be taken on priority for determining the impact on overall rumen methanogenesis.

## Introduction

Globally, livestock are responsible for 15% of anthropogenic greenhouse gas emissions (Gerber et al., 2013). Methane is a significant greenhouse gas (Yusuf et al., 2012), and its atmospheric concentration with an average yearly acceleration of 10 ppb reached 1,890 ppb (Dlugokencky, 2021). Enteric fermentation is one of the primary contributors to atmospheric methane. About 87 to 90 teragrams of methane is generated annually by enteric fermentation (Chang et al., 2019). In addition to contributing to global warming, enteric methane is responsible for a substantial loss of 2%–12% of dietary energy (Johnson and Johnson, 1995). Each liter of enteric methane emission carries 39.5 kJ of the dietary energy away from the animal (Guan et al., 2006). Ionophores have been widely used to improve ruminant productivity while simultaneously reducing enteric methane emissions. However, their use in the diet has been prohibited in many countries (EU directive 1831/2003/EEC). An approach that leads to a significant reduction in enteric methane without affecting ruminal fermentation, the host animal, or residual excretion in products would be one of the most plausible.

Use of plant secondary metabolites containing phyto-sources appears to be one of the promising approaches for enteric methane mitigation. The anti-methanogenic property of tannins has been extensively studied in recent years (Puchala et al., 2005; Grainger et al., 2009; Tan et al., 2011; Jayanegara et al., 2015). Tannins, which are polyphenolic metabolites, can modulate rumen fermentation and mitigate enteric methane emissions (Cieslak et al., 2012; Hassanat and Benchaar, 2013). Based on previous research by our group (Bhatta et al., 2001; Malik et al., 2017a,b; Baruah et al., 2019; Poornachandra et al., 2019; Thirumalaisamy et al., 2022a,b), we have formulated a patent-applied (Indian patent application 201,941,004,992) tanniferous anti-methanogenic supplement “Harit Dhara.” The product was formulated primarily using phyto-sources, furnishing the condensed and hydrolysable tannins in a 3:1 ratio in the final product. We hypothesized that the supplementation of anti-methanogenic product in the straw and concentrate-based diet when included at the appropriate level would mitigate the enteric methane emissions and rumen microbial composition without any detrimental implications on the rumen fermentation. This study aimed to (1) investigate the impact of variable levels of supplementation of anti-methanogenic product on *in vitro* methane production and optimize the most promising inclusion level for animal studies; and (2) to ascertain the impact of anti-methanogenic product supplementation on enteric methane emissions, whole rumen metagenome and ruminal fermentation.

## Materials and methods

### Tannin assays

For the tannin assays, samples of anti-methanogenic supplement in triplicate were ground to a fine powder in a Cyclotec mill (Foss, Denmark). About 20 mg of sample was extracted in 10 ml of aqueous acetone (70%), vortexed for 5 min, and the supernatant (10 μl) was taken for the immediate tannin assay. The tannin assay and the phenol and tannin content in the extract were determined by the Folin–Ciocalteu method (Makkar, 2003) using polyvinylpolypyrrolidone (PVPP). Condensed tannin in the samples was determined following the butanol–HCl–iron method (Makkar, 2003). Total phenols and tannin were expressed as tannic acid equivalents, whereas condensed tannin was leucocyanidin equivalent. The concentration of hydrolysable tannin in the samples was determined by the difference between total and condensed tannin.

### Catechin derivatives

About 0.5 g of sample was weighed and placed in a 50 ml centrifuge tube. After that, 25 ml of diluent (70% methanol) was added, vortexed for 10 min, and kept in a water bath at 70°C for 30 min. Subsequently, the solution was brought to room temperature and made up to a final volume of 50 ml with the diluent. The solution was vortexed and filtered through a 0.45 μm syringe filter into the autosampler vial for HPLC analysis. The individual constituents of the tannin were estimated on the HPLC (1,260 Infinity II, Agilent) using a Zorbax Eclipse C18 column (4.6 × 250 mm × 5 μm), UV detection at 270 nm, and gradient elution. Acetonitrile was used in the mobile phase. The standard (1,000 ppm) of individual tannin constituents (Sigma Aldrich, >99% purity) as an internal reference was injected into the HPLC-DAD.

### Experiment I-*in vitro*

An *in vitro* study was initially conducted to investigate the impact of the anti-methanogenic supplement named “*Harit Dhara*” (HD) on methane production and to optimize the level of supplementation in the diet. A basal diet consisting of finger millet (*Eleusine coracana*) straw and concentrate mixture in a 1:1 ratio was formulated for the *in vitro* evaluation. The concentrate mixture was formulated with maize grain (320 g/kg), soybean meal (130 g/kg), groundnut cake (120 g/kg), wheat bran (400 g/kg), mineral mixture (20 g/kg), and salt (10 g/kg). The anti-methanogenic supplement was added at 2 (HD_2_), 5 (HD_5_), and 8% (HD_8_) levels of the basal diet, whereas in control (CON), there was no supplementation of the HD.

#### Chemical composition

The dry matter content of the samples was determined in accordance with AOAC (2012) at 100°C for 12 h, and the dried samples were ground using a Cyclotec mill. For determining total ash, the samples were initially burned in crucibles on a hot plate before being transferred to a muffle furnace at 550°C for 4 h (AOAC, 2012). Organic matter (OM) was calculated by subtracting the total ash from the sample’s initial dry weight and expressing the result as a percentage. Crude protein (CP, N × 6.25) was determined using an automatic nitrogen analyzer (Gerhardt, Germany) in accordance with AOAC (2005). The samples’ crude fiber (*CF*) was analyzed with an automatic fiber analyzer (Fibretherm FT12, Gerhardt, Germany) in accordance with AOAC (2005), whereas the fiber fractions were determined according to Van Soest et al. (1991). As per AOAC (2005), the ether extract (EE) was determined using Soxtherm (Gerhardt, Germany).

#### Total gas and methane

Two adult male fistulated Holstein-Friesian cattle (BW ± SE, 500 ± 15 kg) were used as donors for the ruminal fluid, which was then used as a source of microbial inoculum. The cattle were fed on finger millet straw and concentrate-based diet (1:1). About 200 mg sample was quantitatively transferred to a 100-ml glass syringe (Haeberle, Germany). The buffer medium consisted of macro and micro mineral solutions, buffer, and resazurin was prepared on the previous day of incubation and preserved in a water bath at 39°C with intermittent flushing of carbon dioxide and stirring. Both the solid and liquid fractions (1:2) were ensured while collecting the rumen fluid on the day of incubation The solid and liquid fractions were brought to the laboratory in a thermos flask at 39°C within the 10 min of collection. The rumen content was squeezed through double layers of muslin cloth into a prewarmed thermos flask while slowly flushing the carbon dioxide. About 30 ml of premixed buffer medium consisting of rumen fluid and medium in a 1:2 ratio was dispensed using an automatic dispenser (Eppendorf Varispenser, 50 ml) into the glass syringe through the silicon tube fitted to the nozzle. For each sample, three replicates were taken and the incubation was repeated three times. Thus, a total of nine observations for each sample/treatment were recorded. The incubation was performed in a Hohenheim type water bath at 39°C for 24 h with periodical automatic shaking. In each incubation, three glass syringes containing buffer medium and rumen fluid without feed sample and three Hohenheim hay standard were placed as blanks and positive controls, respectively. After 24 h, the incubation was terminated and the final piston position was recorded. The syringes were placed on the ice in a big plastic tumbler and the gas was transferred to 10 ml pre-evacuated glass vials with the help of airtight gas syringes. The volume of gas produced was calculated by considering the initial and final piston position as well as the blanks. The gas samples collected in glass vials were presented to the gas chromatograph (Agilent 7890B) using airtight Haeberle glass syringes. The analysis of methane using gas chromatograph was performed by uphold the conditions described previously by Malik et al. (2017a, 2019). The dry matter digestibility (DMD), as described by Menke et al. (1979), was used to determine the effect of graded supplementation of HD on the diet fermentation.

#### Statistical analysis

Data from the *in vitro* study was examined for gaussian normal distribution using the D’Agostino-Pearson normality test at the 0.05 alpha level in GraphPad Prism version 9 (GraphPad Software, San Diego, United States). Total gas, methane, and dry matter digestibility data were analyzed in SPSS version 21.0 (IBM® SPSS, USA) using one-way analysis of variance (ANOVA) with the model.


$$Y_{ij=}\mu +A_{i}+\varepsilon_{ij}$$

Yij represents an individual observation, represents the mean of the population, Ai represents the effect of the treatment, and ij indicates experimental error. Using Tukey’s *post-hoc* procedure, the difference between the means was examined and deemed significant at *p* ≤ 0.05.

The linear effect of the variable doses of anti-methanogenic supplement Harit Dhara on *in vitro* total gas, methane, and dry matter digestibility was determined using the orthogonal polynomial contrast coefficient in SPSS version 21.0.

### Experiment II-animal study

#### Animal feeding and management

After obtaining prior approval (NIANP/IAEC/1/2019) from the Institute Animal Ethics Committee (IAEC), a study in sheep was conducted in strict accordance with the IAEC’s procedures for animal care and sample collection. Twelve adult male *Mandya* sheep (BW ± SD, 30.7 ± 2.05) were randomly divided into two groups of six animals each. Animals were fed *ad libitum* a total mixed ration (TMR) of finger millet straw and concentrate mixture in equal proportions, either without (CON) or with the anti-methanogenic supplement Harit Dhara (HD treatment). Based on the *in vitro* studies, the level of HD supplementation (5% of DMI) was fixed for the sheep feeding. The sheep were housed in a well-ventilated, concrete shed with individual feeding and watering facilities. Before the commencement of the experiment, the animals were dewormed with an anthelminthic (Fenbendazole @5 mg/kg BW). Animals had access to clean drinking water throughout the day.

#### Daily methane emission and nutrient digestibility

Following a preliminary feeding of sheep on CON and HD treatment diets for 30 days, the daily enteric methane measurement was performed using sulfur hexafluoride (SF_6_) tracer technique (Berndt et al., 2014). The brass permeation tubes, which served as the source of SF_6_ gas in the rumen during methane measurement, were charged with 780 ± 52.2 mg pure gas. The charged tubes were calibrated for 70 days at 39°C and the SF_6_ release rate was recorded. Ten days prior to the methane measurement, the calibrated tubes were inserted into the sheep rumen. Throughout the duration of the methane measurement (7 days), a PVC canister to collect background gas samples was hung daily on the iron wire mesh fixed to the cement wall on the east side of the shed. The male and female connectors (Swagelok), capillary tube (Supelco, ID 1/16), teflon tube, and air filter were assembled into the halter and connected to the PVC canister as described by Williams et al. (2014). Individual vacuumed PVC canisters (>90–95 kPa) were tied to each sheep for 24 h to collect breath samples. The timings for the tying and removal of canisters were maintained throughout the duration. After 24 h, the canister was removed from the sheep and the final pressure was measured with a digital pressure meter (Leo 2, Keller). The breath and background samples in the canisters were diluted by two to three times with high-purity N_2_ gas, and the dilution pressure was measured to estimate the dilution factor. Using an airtight glass syringe (Hamilton, 1 ml), successive subsamples were collected from canisters containing diluted breath samples. The samples were then analyzed using a gas chromatograph. For the analysis of methane and SF_6_, a gas chromatograph (GC 2010 plus, Shimadzu, Japan) equipped with flame ionization and electron capture detectors was used under the same circumstances as previous studies (Malik et al., 2021; Thirumalaisamy et al., 2022b).

Methane (ppm) and SF_6_ (ppt) concentrations were calculated using the canister pressures at the beginning, end, and after N_2_ dilution (Lassey et al., 2014). The local elevation and atmospheric pressure at the study site (Thirumalaisamy et al., 2022b) were also taken into consideration while calculating the gas concentration.


$$\left[G_{S}\right]=\frac{90-\tau_{f}}{\tau_{e}-\tau_{s}}\times \left[G_{A}\right]$$

G_S_ was the concentration of methane (ppm) or SF_6_ (ppt), 𝜏_f_ (kPa) was the final pressure after N_2_ dilution, 𝜏_s_ (kPa) was the post sampling pressure, 𝜏_e_ was the initial canister pressure, and G_A_ was the concentration of methane (ppm) or SF_6_ (ppt) in the diluted samples.

Daily enteric methane emissions were calculated as per (Moate et al., 2014).


$$R_{CH4}=R_{SF6}\frac{{\left[CH_{4}\right]}_{M}-{\left[CH_{4}\right]}_{BG}}{{\left[SF_{6}\right]}_{M}-{\left[SF_{6}\right]}_{BG}}\times \frac{MW_{CH_{4}}\;}{MW_{SF_{6}}}\times 1000$$

R_CH4_ was CH_4_ emission (g/d), R_SF6_ was the SF_6_ release rate from permeation tubes (mg/d), [CH_4_]_M_-[CH_4_]_BG_ was the methane concentration in the samples and background, [SF_6_]_M_-[SF_6_]_BG_ was the SF_6_ concentration in the samples and background, and MW_CH4_ and MW_SF6_ represented the molecular mass of the respective gases.

After 30 days of feeding, a seven-day digestibility trial was conducted concurrently with daily methane measurements. The daily total mixed ration (TMR) offered, feed refusals, and fecal output of each individual sheep in the CON and HD treatment groups were recorded. Representative samples of TMR and feed refusals were obtained and dried at 80°C for 24 h. The DMI (g/d) was determined by subtracting the daily feed refusals from the feed offered. Before drawing a representative sample, the total amount of feces voided by each sheep over a period of 24 h was transferred to a large plastic container and thoroughly mixed. One aliquot (1/100th) was taken from the mixed feces for dry matter estimation in a hot air oven at 100°C, while another aliquot (1/1000th) was preserved in 25% sulfuric acid. The chemical constituents in the dried feed, refusals, and feces samples were analyzed using the procedure outlined in the section on chemical analysis. The digestibility coefficient of nutrients was calculated using the following equation.


$$\;\mathrm{Digestibility}\;\mathrm{coefficient}=\frac{Nutrientintake-Excretion\;of\;nutrient}{Intake\;of\;nutrient}$$

#### Ruminal fluid collection

Rumen fluid samples were collected at the completion of the methane measurement study (38th day of feeding). The samples were collected 3 h post-feeding from the individual sheep with the help of 1 m perforated nylon stomach tube connected to an airtight collection vessel and handheld vacuum pump (Malik et al., 2015; Thirumalaisamy et al., 2022a,b). To avoid saliva contamination, the first 30 ml of ruminal fluid was discarded, and then 45 ml of rumen fluid was collected and strained through a single layer of muslin cloth for the separation of liquid and solid digesta fractions. The strained rumen fluid was separated into three 15 ml subsets for the isolation of gDNA, ammonia-VFA analysis, and protozoa enumeration. Immediately prior to being transported to the lab, tubes holding the ruminal fluid for gDNA and ammonia-VFA were placed on ice, while ruminal fluid subsets for the protozoal count were transported to the laboratory without being placed on ice. The solid digesta fraction retained in the muslin cloth was squeezed and washed with the buffer consisting of 50 mM tris–HCl, 200 mM NaCl, 5 mM sodium EDTA, and 0.05% triton and re-suspended in the liquid fraction preserved for the genomic DNA isolation.

#### Rumen fermentation

The samples of ruminal fluid were centrifuged at 13,400 rpm for 15 min, and the supernatant was separated into two equal portions. In one portion, 25% metaphosphoric acid was added in a 4:1 (v/v) ratio and stored at-80°C until the VFA analysis. The samples were thawed at room temperature and the VFA concentration was estimated in accordance with Filípek and Dvořák (2009) using a gas chromatograph (Agilent 7890B, Santa Clara, United States). During the analysis, the following GC conditions were upheld (Malik et al., 2021; Thirumalaisamy et al., 2022b): temperature program: 59°C–250°C (20°C/min, 10 min), injector temperature: 230°C, and detector temperature: 280°C.

Individual volatile fatty acids concentration was determined using the following equation:


$$VFA\;con.\;\left(mmol\right)=\frac{\begin{array}{l} Peakareaofsample\;x\;\\ Conc.\;ofstandard\;x\;dilution \end{array}}{Peakareaofstandard}$$

The concentration of ammonia in the second subset of ruminal fluid samples was determined as per Conway (1957). In brief, 1 ml of a boric acid indicator was pipetted into the inner chamber of the disk, while an equivalent volume of saturated sodium carbonate was pipetted into the outer chamber. Approximately 1 ml of ruminal fluid was pipetted directly just outside of the sodium carbonate in the outer chamber. The disk was covered with the lid and left undisturbed at room temperature for 2 h before being titrated with 0.01 N sulfuric acid. Ammonia-N concentration was determined with the following formula:


$$\mathrm{Ammonia}-N\;\left(\mathrm{mg}/\mathrm{dl}\right)=\mathrm{ml}\;\mathrm{of}0.001NH_{2}SO_{4}x14$$

#### Protozoal enumeration

The protozoa in the rumen fluid (third sub-set) transported without putting on the ice were enumerated on the same day following the methodology described by Kamra and Agarwal (2003). Ruminal protozoa enumeration and morphological identification were carried out under a phase-contrast microscope (Nikon Eclipse, Japan) at a 10x objective. Based on the morphology and cilia, the protozoa were categorized into *Entodiniomorphs* and *Holotrichs* in accordance with Hungate (1966). The numbers of protozoa in the rumen fluid from the individual sheep in CON and HD treatment groups were calculated using the following equation:


$$N=\frac{n\times A\times D}{a\times v}$$

Where, *N* was number of protozoa (cells) per ml of rumen fluid, n was the average cell count per microscopic field, A was the area of the slide on which the diluted rumen fluid sample was spread, D was the dilution, a was the area of the microscopic field, and v was the volume of rumen fluid in the cavity. The protozoal numbers were expressed as ×10^5^ or ×10^4^ cells/ml.

#### Blood biochemical profile

At the end of the methane measurement trial (38th day), the blood samples (5 ml) were collected from the jugular vein in heparinized tubes. Total protein, albumin, globulin, minerals (Ca, P, Mg) and enzymes (alkaline phosphatase, alanine aminotransferase, glutamyl transferase, and creatine kinase) were analyzed in an automatic blood analyzer (VetScan 2, Abaxis, Germany) following the manufacturer’s instructions.

#### Statistical analysis

Data from the animal traits was examined for gaussian normal distribution using the D’Agostino-Pearson normality test at the 0.05 alpha level in GraphPad Prism version 9 (GraphPad Software, San Diego, CA, United States). Data on the intake, nutrient digestibility, daily enteric methane emissions, fermentation, and protozoal counts between the CON and HD treatment groups were analyzed using the independent sample t test in SPSS version 21.0 (IBM® SPSS, United States). The Leven’s test was used to determine the significance of the comparison of the group means at a confidence level of 95%. The principal component analysis (PCA) biplot was constructed by considering methane as a dependent variable and regressing the VFA, protozoa and organic matter intake data for the weightage of the individual parameter on the dependent variable.

### DNA isolation

In order to allow feed particles to settle, the ruminal fluid samples were initially spun at 1,000 rpm for 5 min. The supernatant was transferred to a new tube and kept at-80°C until the gDNA was isolated. The samples were initially centrifuged at 13,400 rpm for 10 min at 4°C, and the dense pellet was retained while the supernatant was discarded. The pellet was dissolved using 1 ml of lysis buffer by gentle pipetting. The contents were transferred to a 2 ml sterile screw cap tube with an O-ring (BioSpec, United States) containing pre-sterilized zirconia beads weighing 0.5 g (0.1 mm; BioSpec, United States). The RBB + C technique (Yu and Morrison, 2004) was used to isolate gDNA from samples of ruminal fluid. The quality of the gDNA was determined using 0.8% agarose gel electrophoresis, and the concentration was determined using a Qubit 4.0 (ThermoFisher Scientific, Waltham, United States).

### Total methanogens quantification

The relative abundance of total methanogen in the CON and HD treatment group was determined by qPCR (Thermo Fisher Scientific, United States) using SsoFast^TM^ Eva Green® Supermix (Bio-Rad Laboratories Inc., CA, United States). The mcrA forward (TTC GGTGGATCDCARAGRGC3) and reverse (GBARGTC GWAWCCGTAGAATCC) described (Denman et al., 2007) were used for the amplification of total methanogens. In brief, 1 ng of total genomic DNA, 0.25 mM of each forward and reverse primer, and 1X Eva Green Supermix were mixed in a reaction volume of 10 ml. The qPCR conditions were upheld as described previously (Thirumalaisamy et al., 2022a). The relative changes in the abundance of total methanogens were determined by the 2^−ΔΔCT^ method. Total methanogens quantification between CON and HD treatment group were compared using *t*-test.

### WGS library preparation and paired end sequencing

All gDNA samples were sent to an external sequencing facility (Clevergene Biocorp, Bangalore, India) for Illumina NextSeq500 metagenome sequencing. To generate an average fragment of 350 bp, DNA shearing was performed using a Covaris M220 (Covaris®) with the following settings: duty factor 20%, peak/display power of 50, cycle/burst 200, temperature 20°C, and time 65 s. End Repair Mix was used to perform end repair on the produced fragments. According to the manufacturer’s instructions, the ligated products were size-selected using AMPure XP beads and amplified using the index primers (Illumina True Seq Nano DNA Library Prep Kit). Following the ligation of indexing adaptors to the ends of DNA fragments, PCR-enriched libraries were analyzed on a 4,200 Tape Station System (Agilent Technologies). The libraries were loaded onto NextSeq500 for cluster generation and sequencing, and 150 bp paired-end reads (2 × 150 bp) were obtained.

### Structural and functional annotation of rumen metagenome

The MG-RAST Metagenomics Analysis server was used to annotate individual datasets under the project id PRJNA885963. The conventional MG-RAST pipeline was used to perform the quality control and merge of the paired-end reads. The low-quality sequence reads were iterated based on a Phred quality score of 15. The artificially replicated reads were eliminated and the sequences were checked for host contamination using the *B. taurus* UMD v3.0 database. BLAT (Kent, 2002) and the M5nr database (Wilke et al., 2012) were used to taxonomically classify the reads. A minimum *E-value* cut-off of 1 × 10^−5^ was used, while the minimum percentage identity cut-off was set at 60%. The reads were labeled as “unclassified” if they did not meet the aforementioned criterion at the selected taxonomic level. Sequences that could not be attributed to a taxonomic unit were labeled “No Hits.” The FragGeneScan method (Rho et al., 2010) was used to name genes from the metagenomic profile generated by MG-RAST (Wilke et al., 2013), and UCLUST was used to cluster the predicted protein sequences (Edgar, 2010). The longest sequence from each cluster was chosen as the representative sequence for matching with the M5nr reference database (Wilke et al., 2012). The analysis page of MG-RAST was used to export the annotated results and taxonomic affiliations of bacteria and archaea obtained from the RefSeq database. Thereafter, the abundance tables of taxonomic assignments for the bacteria and archaea were uploaded separately into MicrobiomeAnalyst (Chong et al., 2020) for statistical computation and visualization of the metagenome data.

### Differential abundance estimation and statistical analysis

The initial filtering of the metagenome data in MicrobiomeAnalyst was performed using the following cut-off values: minimum abundance counts of 4, prevalence in samples of 20%, and a low variance filter of 10%. The data was normalized using rarefication to the minimum library size, and the abundance and evenness of the microbiome composition were evaluated using Shannon’s diversity index. Principal coordinate analysis (PCoA) was used to depict the beta diversity based on Bray–Curtis dissimilarity matrices, and Permutational Analysis of Variance (PERMANOVA) was used to calculate the statistical difference. In addition, based on the Bray–Curtis dissimilarity matrices, and Permutational Analysis of Variance (PERMANOVA) in the MicrobiomeAnalyst, the coefficient of determination (R^2^) was determine for estimating the proportion of variance. The taxonomic profiles of bacteria and archaea between CON and HD treatment groups at the phylum and genus level were compared using t test. The differential abundance of taxonomical assignment at the genus level was estimated using DeSeq2 (Love et al., 2014) on the raw counts. The fold changes and the corresponding *p* values of the bacterial and archaeal differential abundance were depicted in the volcano plot.

### *De-novo* assembly and annotation

The whole metagenome data set was assembled using Megahit (version 1.2.9) on the KBase portal (Arkin et al., 2018). The contigs were used for predicting prokaryotic genes and translated into amino acids using Find prokaryotic genes (version 2.2) and translated to protein module (version 21.0.2) with translation table 11 containing bacterial, archaeal, and plant plastids, respectively, in the CLC Genomics workbench (version 21.0). The amino acid sequences in the FASTA file were matched to C_Genus_Prokaryotes in Ghost Koala (Kanehisa et al., 2016) and summarized in R (version 4.0.2). The KOs identified in the CON and HD treatment groups were mapped using the KEGG reconstruct pathway tool and visualized the methane metabolism pathway. Further, the KO table was filtered based on the total minimum count of 10 in at least one of the groups, and the differential abundance of the KOs was estimated using DESeq2. The KOs related to the methanogenesis pathways were filtered from the DESeq output.

## Results

### Tannin-catechin derivatives

Tannin analysis revealed that the formulated product contains 22.1% tannic acid (Cyanidin equivalent). The anti-methanogenic supplement primarily contains condensed tannins (75%), while hydrolysable tannin accounts for 25% of the total tannic acid in the supplement. The condensed to hydrolyzable tannins ratio was 3.12:1 (Table 1). HPLC analysis of catechin derivatives revealed that epigallocatechin (8.23%) was most abundant, followed by epicatechin (0.61%) and gallic acid (0.55%). Other derivatives, such as ellagic acid, catechin and epicatechin, and gallate, accounted for less than 0.2% of the derivatives (Table 1).

**Table 1:** Tannin assay and catechin derivatives in anti-methanogenic supplement.

Tannin assay (%)					
Phenol	Non-tannin phenol	Tannic acid	CT	HT	CT: HT
23.06	0.96	22.09	16.73	5.36	3.12:1
**Catechin derivatives (%)**					
Catechin	Epicatechin	Gallic acid	Ellagic acid	Epigallocatechin	Epicatechin gallate
0.13	0.61	0.55	0.17	8.23	<0.01

### *In vitro* total gas and methane production

The chemical composition of the basal diet and anti-methanogenic supplement is presented in Supplementary Table 1. The results of the *in vitro* experiments (Table 2) revealed a significant decrease in total gas (TG) production at the highest level (HD_8_) of anti-methanogenic agent supplementation. In a similar manner, the dry matter digestibility (DMD) decreased (*p* < 0.0001) when an anti-methanogenic agent was added at a level of 8%. Data on methane production revealed a significant decrease (*p* < 0.001) across all three levels (HD_2_, HD_5_, and HD_8_) of anti-methanogenic supplementation compared to the control (*p* < 0.001). The difference in methane production between the HD treatments was also significant (*p* = 0.008). The addition of anti-methanogenic product at varying concentrations reduced (*p* < 0.0001) the proportion of methane in total gas (Table 2). Overall, there was a tendency of linear reduction in the total gas, methane, DMD and methane as percent of total gas due to the variable doses of the anti-methanogenic supplement.

**Table 2:** Effect of anti-methanogenic supplement on *in vitro* gas production and DMD.

Attributes	CON	HD_2_	HD_5_	HD_8_	SEM	*P*	Linear, *P*
Total gas (ml/g)	236^b^	234^b^	229^b^	215^a^	2.06	<0.0001	<0.0001
Methane (ml/g)	42.0^c^	35.2^b^	33.8^b^	29.1^a^	1.15	<0.0001	<0.0001
Methane (% of TG)	18.3^c^	16.2^b^	15.7^ab^	14.7^a^	0.321	<0.0001	<0.0001
DMD (%)	63.8^b^	62.9^b^	62.3^b^	60.7^a^	0.274	<0.0001	<0.0001
Methane reduction* (ml/g)	-	15.9^a^	19.3^a^	30.6^b^	2.26	0.008	<0.0001

CON, basal diet + 0 *Harit Dhara*; HD_2,_ basal diet + 2% *Harit Dhara*; HD_5_, basal diet + 5% *Harit Dhara*; HD_8_, basal diet + 8% *Harit Dhara*. DMD, dry matter digestibility; dDM, digestible dry matter; TG, total gas. Mean values bearing different superscripts in a row differ significantly at 5% level.

* Reduction is in percent.

### Methane emission and nutrient digestibility

The chemical composition of the basal diet is presented in Supplementary Table 2. Daily enteric methane measurement (g/d) in sheep revealed a significant reduction (*p* = 0.0002) in the anti-methanogenic supplemental group (17.2 g/d) compared to the CON group (21.9 g/d). Similarly, the anti-methanogenic agent in the HD treatment group also decreased (*p* = 0.0008) methane yield (g/kg DMI) as compared to the CON group. The nutrient intake and digestibility data (Table 3) did not reveal any negative effects of the anti-methanogenic supplement on the intake or nutrient digestibility in sheep.

**Table 3:** Effect of *Harit Dhara* supplementation on nutrient intake, digestibility, and methane emissions in sheep.

Attribute	CON	HD	SEM	*P*
Body weight (kg)	30.8	30.7	0.593	0.894
**Intake (g/d)**				
Dry matter	943	941	1.82	0.551
Organic matter	868	877	5.43	0.452
Crude protein	116	117	0.69	0.501
Neutral detergent fiber	385	389	2.40	0.471
Acid detergent fiber	294	297	1.80	0.458
**Dig. nutrient intake**				
Dry matter	595	611	13.9	0.570
Organic matter	560	575	12.9	0.596
Crude protein	77.8	79.6	1.59	0.591
Neutral detergent fiber	155	166	8.84	0.571
Acid detergent fiber	111	119	7.03	0.586
**Methane emissions**				
Daily methane (g/d)	21.9	17.2	1.17	0.0002
Methane yield (g/kg DMI)	23.2	18.2	0.489	0.0008

CON, control group (no *Harit Dhara* supplementation); HD, treatment group (*Harit Dhara* supplementation @ 5% basal diet); DMI, dry matter intake; DDMI, digestible dry matter intake; DCP, digestible crude protein intake; DADF, digestible acid detergent fiber; DNDF, digestible neutral detergent fiber; CH_4,_ methane; SEM, standard error of mean. Mean values bearing different superscripts in a row differ significantly at 5% level.

### Rumen fermentation and protozoa

Similar to nutrient intake and digestibility, total VFA production (mM) did not differ between the CON and HD treatment groups (*p* = 0.062). In addition, the production of specific VFAs also did not differ between the CON and HD treatment groups. However, the propionate production (mM) in the HD treatment group was higher (*p* = 0.007) than in the CON group. The amount of ammonia nitrogen (mg/dL) was higher in the HD treatment group (*p* = 0.026) than in the control group (CON). Due to the addition of an anti-methanogenic agent, the number of protozoa were significantly reduced in the HD treatment group (Table 4). The inclusion of the anti-methanogenic supplement (HD treatment group) led to a significant reduction in the total number of *Entodinimorphs* and *Holotrichs* protozoa.

**Table 4:** Effect of *anti-methanogenic* supplement on rumen fermentation and protozoa in sheep.

Attributes	CON	HD	SEM	*P*
TVFA (mmol)	67.9	75.9	2.18	0.062
Ammonia N (mg/dl)	10.8	9.57	0.304	0.026
**VFA (mmol)**				
Acetate	47.8	51.2	1.52	0.285
Propionate	10.9	13.0	0.432	0.007
Butyrate	7.07	9.07	0.526	0.051
Isobutyrate	0.69	0.80	0.046	0.227
Valerate	0.51	0.61	0.033	0.124
Isovalerate	0.88	1.24	0.102	0.068
A/P	4.19	4.24	0.115	0.835
**Protozoa**				
Total (× 10^5^ cells/ml)	5.71	0.79	0.811	<0.0001
Entodiniomorphs (× 10^5^ cells/ml)	5.52	0.79	0.788	<0.0001
Holotrichs (× 10^4^ cells/ml)	1.98	nd	0.389	0.004

CON, control group (no *Harit Dhara* supplementation); HD, treatment group (*Harit Dhara* supplementation @ 5% basal diet); SEM, standard error of mean. Mean values bearing different superscripts in a row differ significantly at 5% level.

**Figure 1:**
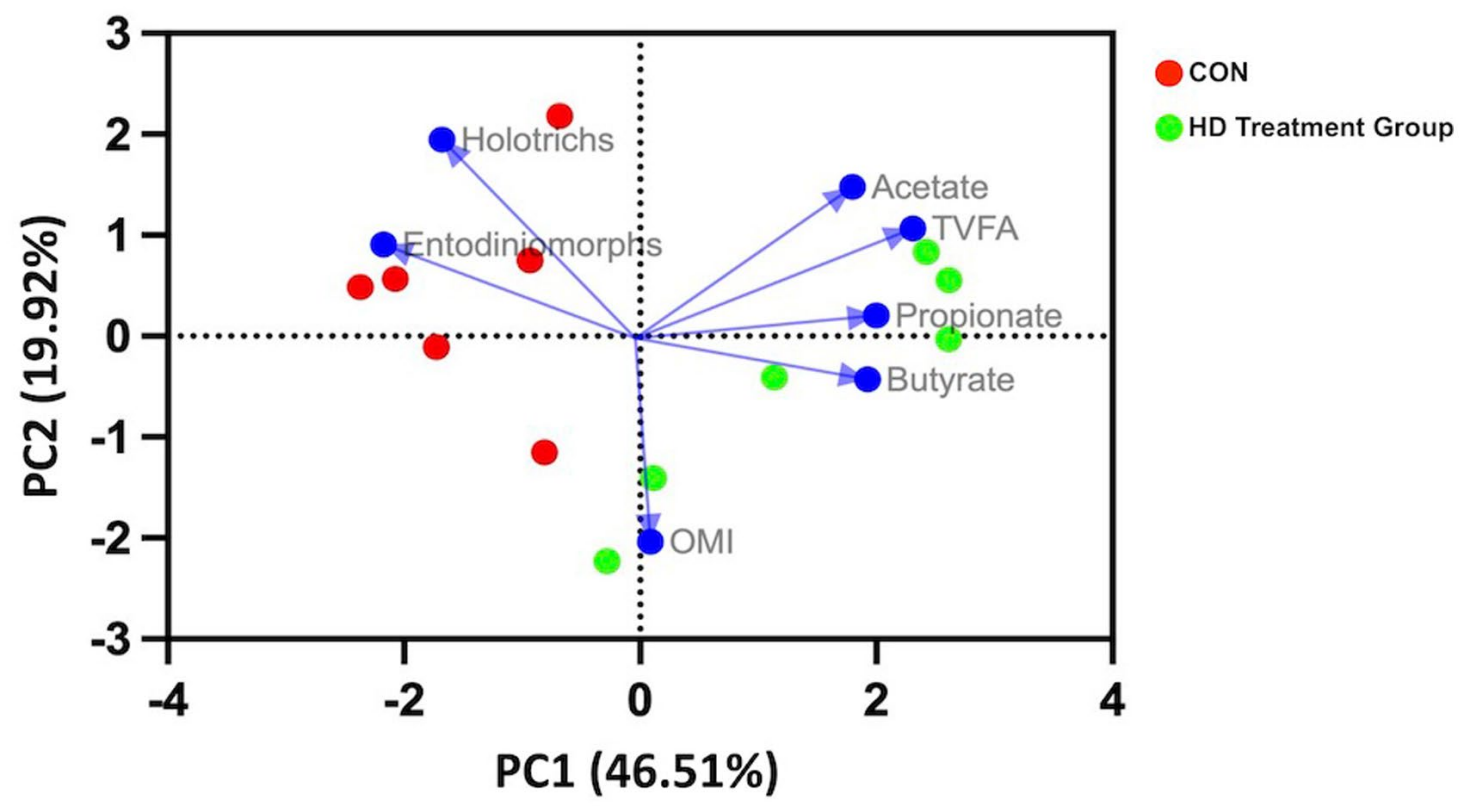
PCA biplot of CON and HD treatment group samples. The samples (points) were distinguished by VFA, protozoa, and organic matter intake (arrows labeled). The first principal component is dominated by VFA, whereas the second principal component is dominated by organic matter intake.

The PCA biplot (Figure 1) plotted considering methane yield as the main attribute revealed that the PC1 had relatively more influence (46.51%) than the PC2 (19.92%). *Entodinimorphs* and *Holotrichs*’ protozoa had a significant impact on methane production. The close angle of *Entodinimorphs* on PC loadings revealed that they were closely correlated with methane production as compared to the *Holotrichs*. The propionic acid had more influence on the PC1, whereas acetate had an impact on PC2 and was found to be positively correlated with the methane yield.

### Blood biochemical profile

Blood analysis revealed comparable enzymatic activity, mineral content, and protein (albumin, globulin, total protein, and blood urea nitrogen) between the CON and HD treatment groups (Supplementary Table 3). Results indicated that the anti-methanogenic supplement did not alter the blood biochemical profile.

### Total methanogens

Results from the study revealed a significant (*p* = 0.016) reduction (−1.46 log fold change) in the total rumen methanogens quantified by qPCR in the HD treatment group as compared to CON (Supplementary Figure 1).

### Rumen whole metagenome

A total of 182,783,020 reads and 18,278,302 ± 583,571 sequences per sample were generated by metagenomic shotgun sequencing of ruminal fluid samples from sheep in CON and HD treatment groups. Each sample yielded an average of 5.85 GB of paired-end read data (range 31.9–44.8 million). A total of 84.69% of the submitted sequences passed the MG-RAST pipeline’s quality control. For taxonomic analysis, each sample’s reads mapped to the RefSeq database were rarefied to 15,452,419 for bacteria and 178,972 for archaea.

**Figure 2:**
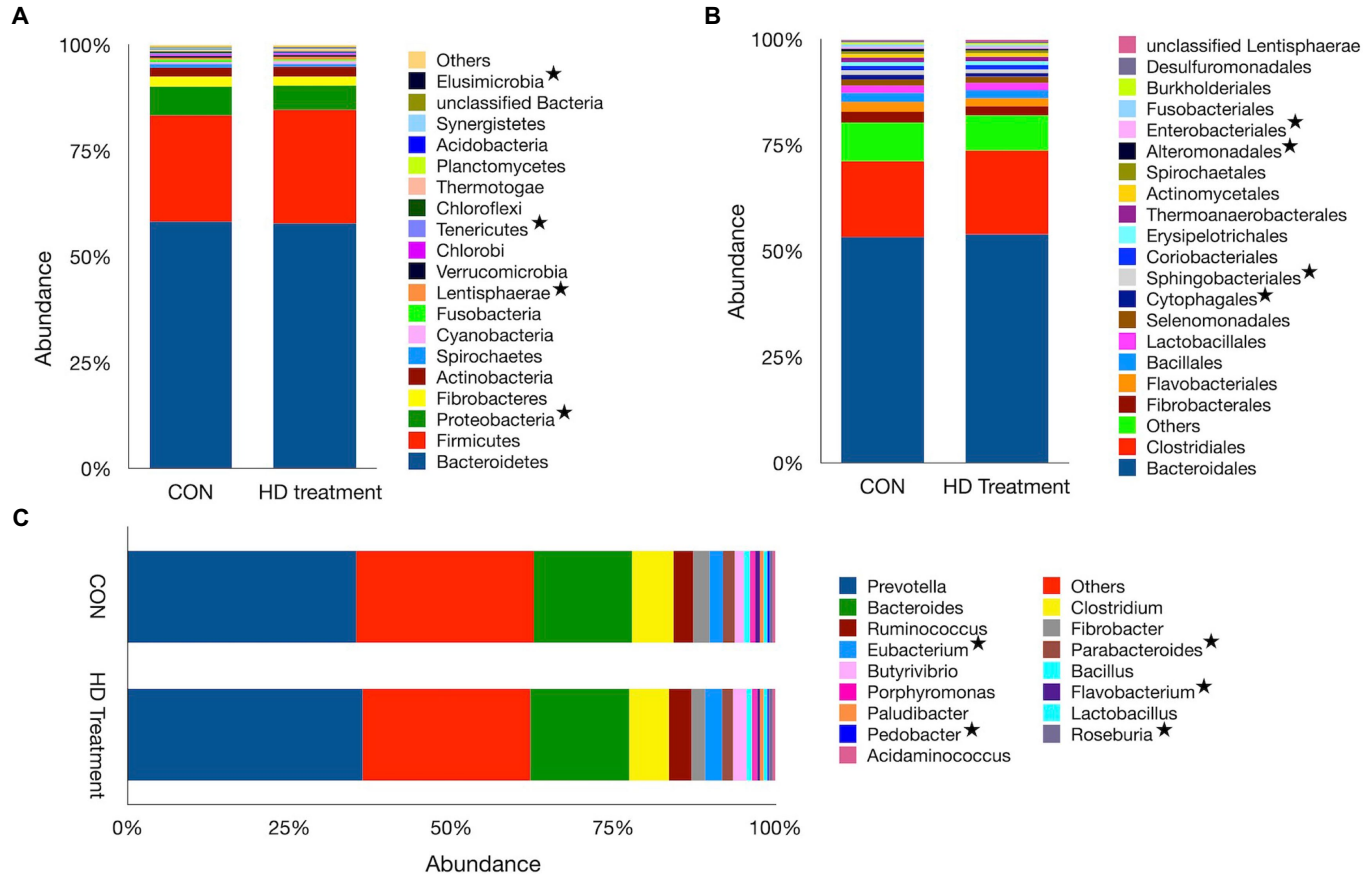
Bacterial profiles at different phylogenetic levels observed in the CON and HD treatment groups. The relative abundances of bacterial taxa at the phylum **(A)**, order **(B)**, and genus **(C)** levels are represented by bar plots. The category “Others” represents taxa with a relative abundance of <1%. Asterisks denote taxa that significantly differ in abundance between the CON and the HD treatment groups.

The shotgun dataset contains 28 bacterial phyla, 110 orders, and 571 genera. *Bacteroidetes* (57.9%), *Firmicutes* (26.1%), *Proteobacteria* (6.21%), *Actinobacteria* (2.31%) and *Fibrobacteres* (2.23%) were the most abundant bacterial phyla, aggregately constituting ~95% of the ruminal bacteria in sheep. The Bacterial phyla dataset revealed similar *Bacteroidetes* (*p* = 0.726), *Firmicute*s (*p* = 0.170), *Fibrobacteres* (*p* = 0.314) and *Actinobacteria* (*p* = 0.181) abundance between CON and HD treatment groups (Supplementary File 1). However, the supplementation of anti-methanogenic agent in the HD treatment group led to a reduction (*p* = 0.013) in the abundance of *Proteobacteria*. Similarly, the abundance of *Tenericutes* (*p* = 0.010) and *Elucimicrobia* (*p* = 0.019) was also adversely affected by the supplement. On the contrary, the abundance of *Lentisphaerae* in the HD treatment (0.67%) was greater (*p* = 0.032) than the CON group (0.47%, Figure 2A). The average *Firmicutes t*o *Bacteroidetes* ratio was comparable between the groups. At the order level, *Bacteroidales* and *Clostridiales,* with a corresponding average abundance of 53.5 and 18.8%, were two prominent orders of the bacteria. The abundances of *Bacteroidales* (*p* = 0.689) and *Clostridiales* (*p* = 0.086) between the CON and HD treatment groups were not significantly different (Figure 2B). However, the minor bacterial orders such as *Cytophagales* (*p* = 0.027), *Sphingobacteriales* (*p* = 0.032), *Alteromonadales* (*p* = 0.030) and *Enterobacteriales* (*p* = 0.037) were differentially distributed between the CON and HD treatment groups. In the present study, *Prevotella* (35.1%), others (26.2%), and *Bacteroides* (14.9%) were three prominent bacterial genera (Figure 2C).

**Figure 3:**
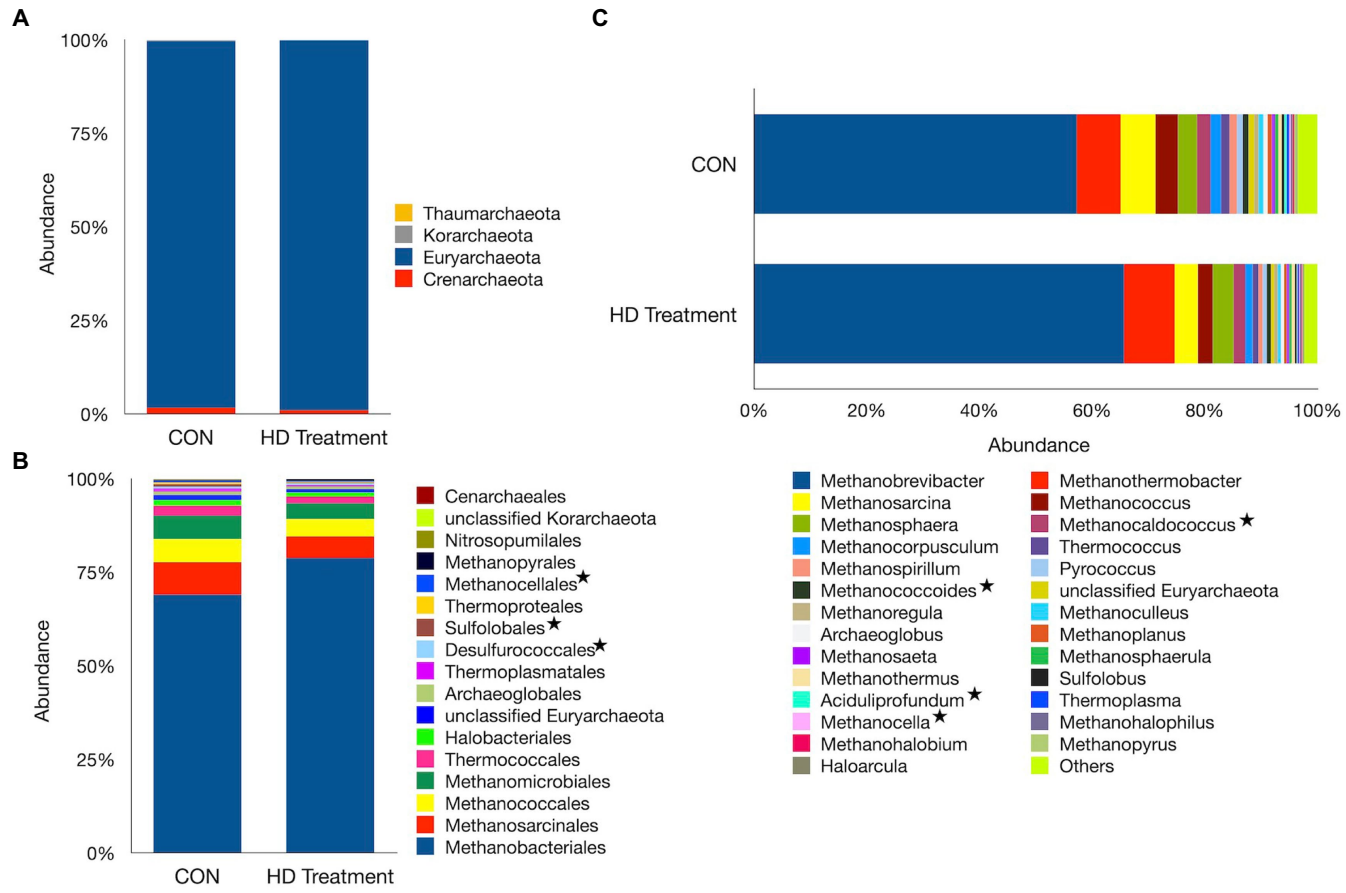
Archaeal profiles at different phylogenetic levels. Bar graphs illustrate the relative abundances of archaeal taxa at the phylum **(A)**, order **(B)**, and genus **(C)** levels. In the plots, the category “Others” represents taxa with relative abundances of <1%. Asterisks denote taxa that differ in abundance between the CON and the HD treatment groups.

On the other hand, 4 phyla, 17 orders, and 54 genera of archaea were identified in the present study in sheep. The *Euryarchaeota* was the most abundant phylum, accounting for 98.4% of the total archaea, while the *Crenarchaeaota* accounted for 1.30% of the total archaea (Figure 3A). The archaea assigned to the *Korarchaeaota* and *Thaumarchaeota* were also identified; however, at an extremely low frequency (0.2%). *Methanobacteriales* (73.9%) and *Methanosarcinales* (7.10%) were the two most abundant archaeal orders, but their distribution did not vary between the groups (Figure 3B). The *Methanococcales* and *Methanomicrobiales* were another two important archaeal orders, each of which was distributed with an individual frequency of ~5% (Supplementary File 1). The abundance of minor archaeal orders such as *Desulforococcales* (*p* = 0.029), *Sulfolobales* (*p* = 0.043), and *Methanocellales* (p = 0.043) differed significantly between the CON and HD treatment groups.

The *Methanobrevibacter* genus, with a similar distribution (*p* = 0.07) between the two groups, constituted the largest fraction (avg. 61.5%) of the total archaea in sheep (Figure 3C). Similarly, the abundance of *Methanosarcina*, with a mean distribution of 5.19%, was not significantly different between groups (*p* = 0.065). The anti-methanogenic supplementation in the HD treatment group significantly decreased the abundances of *Methanocaldococcus* (*p* = 0.011), *Methanococcoides* (*p* = 0.042), *Methanocella* (*p* = 0.043), and *Methanoregula* (*p* = 0.040). In contrast, the abundance of the second largest genus, *Methanothermobacter,* was significantly greater in the HD treatment group (*p* = 0.029).

**Figure 4:**
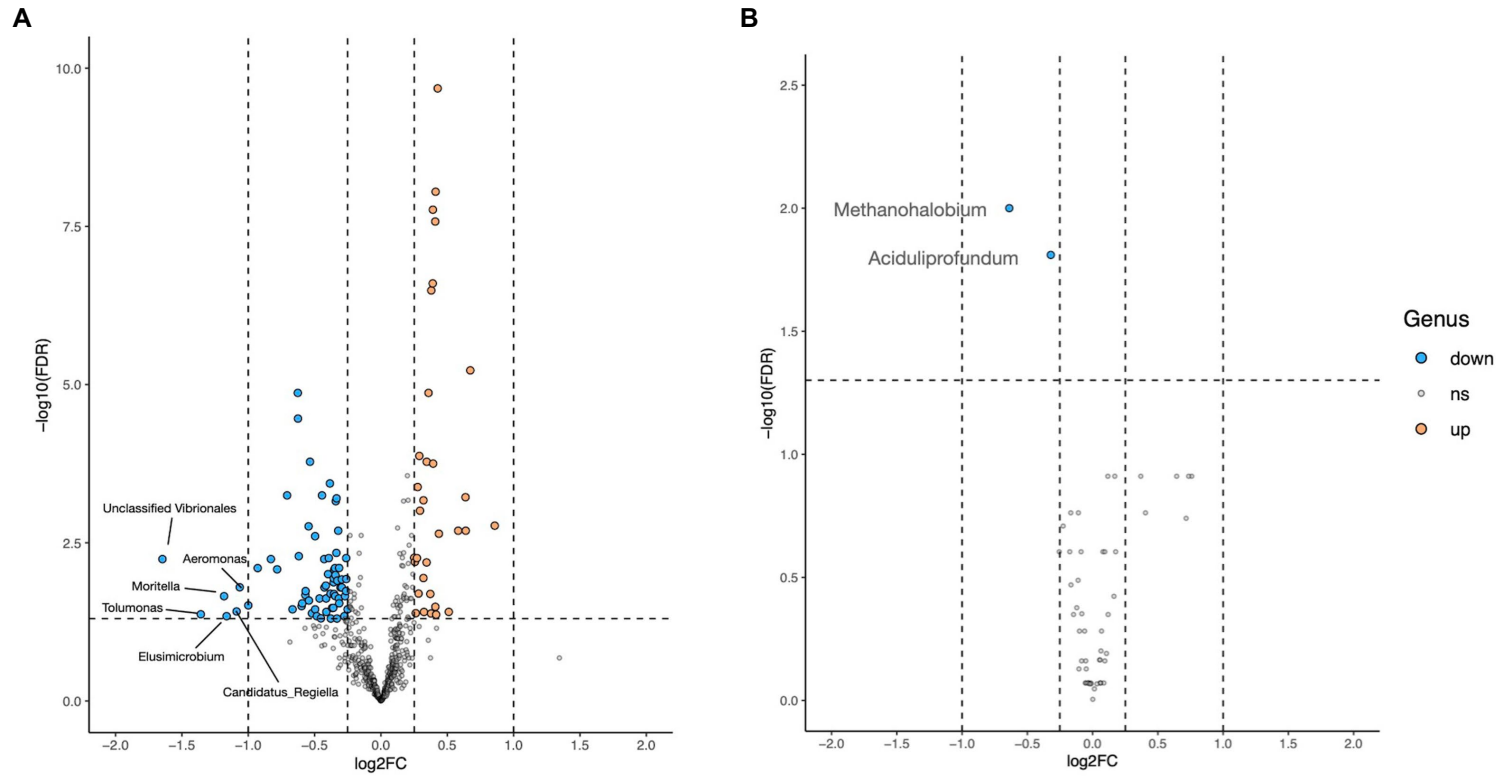
Volcano plot showing the difference in bacterial **(A)** and archaeal **(B)** abundance at the genus level between the CON and HD treatment groups. The blue dot indicates downregulation, the orange dot indicates upregulation, while gray dots depict no change. The log2 fold change is represented on the x-axis represents, while the y-axis represents the-log10 of FDR-corrected *p*-values.

Volcano plot revealed that the abundances of six (*Candidatus Reigiella*, *Elusimicrobium*, *Tolumonas*, *Moritella*, *Aeromonas*, and unclassified *Vibrionales*) among the 571 identified genera were decreased by >2-fold in the HD treatment group (Figure 4). However, no genus was found to be upregulated in the HD treatment group. The volcano plot revealed a two-fold downregulation in *Methanohalobium* and *Aciduliprofoundum* archaeal genera.

### Alpha and beta diversity

**Figure 5:**
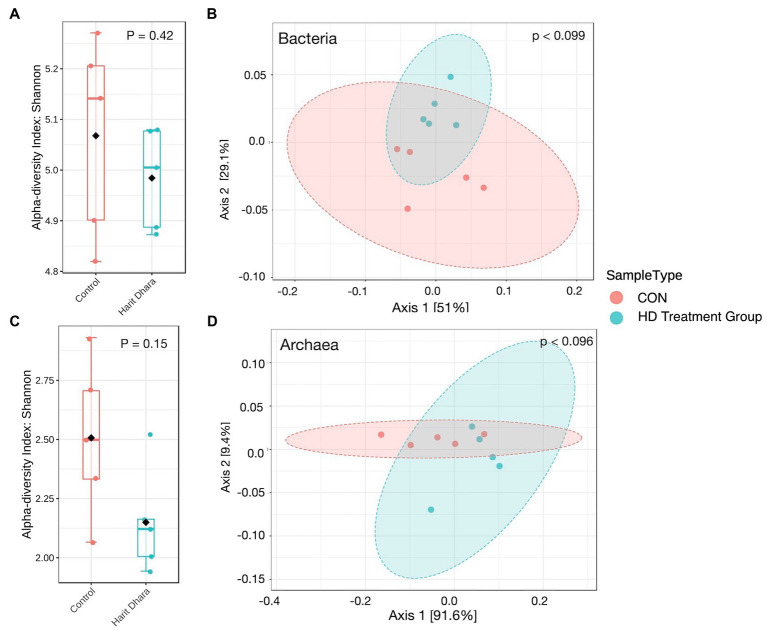
Alpha and beta diversity plots of bacteria **(A,B)** and archaea **(C,D)**.

The diversity and richness of bacterial communities in the CON and HD treatment groups, as measured by the Shannon index (Figure 5A), were not significantly different (*p* = 0.42). Similarly, there was no difference in the diversity and richness of archaeal communities between the two groups (*p* = 0.15). The Bray–Curtis dissimilarity visualized using PCoA plots revealed comparable bacterial community composition between the two groups (*p* = 0.099, Figure 5B). Similar patterns were observed in the archaeal community structure between the CON and HD treatment groups (Figures 5C,D; *p* = 0.096). The coefficient of determination (R^2^) was non-significant between the CON and HD treatment groups for bacteria (*r* = 0.22) and archaea (*r* = 0.30).

### KEGG analysis

**Figure 6:**
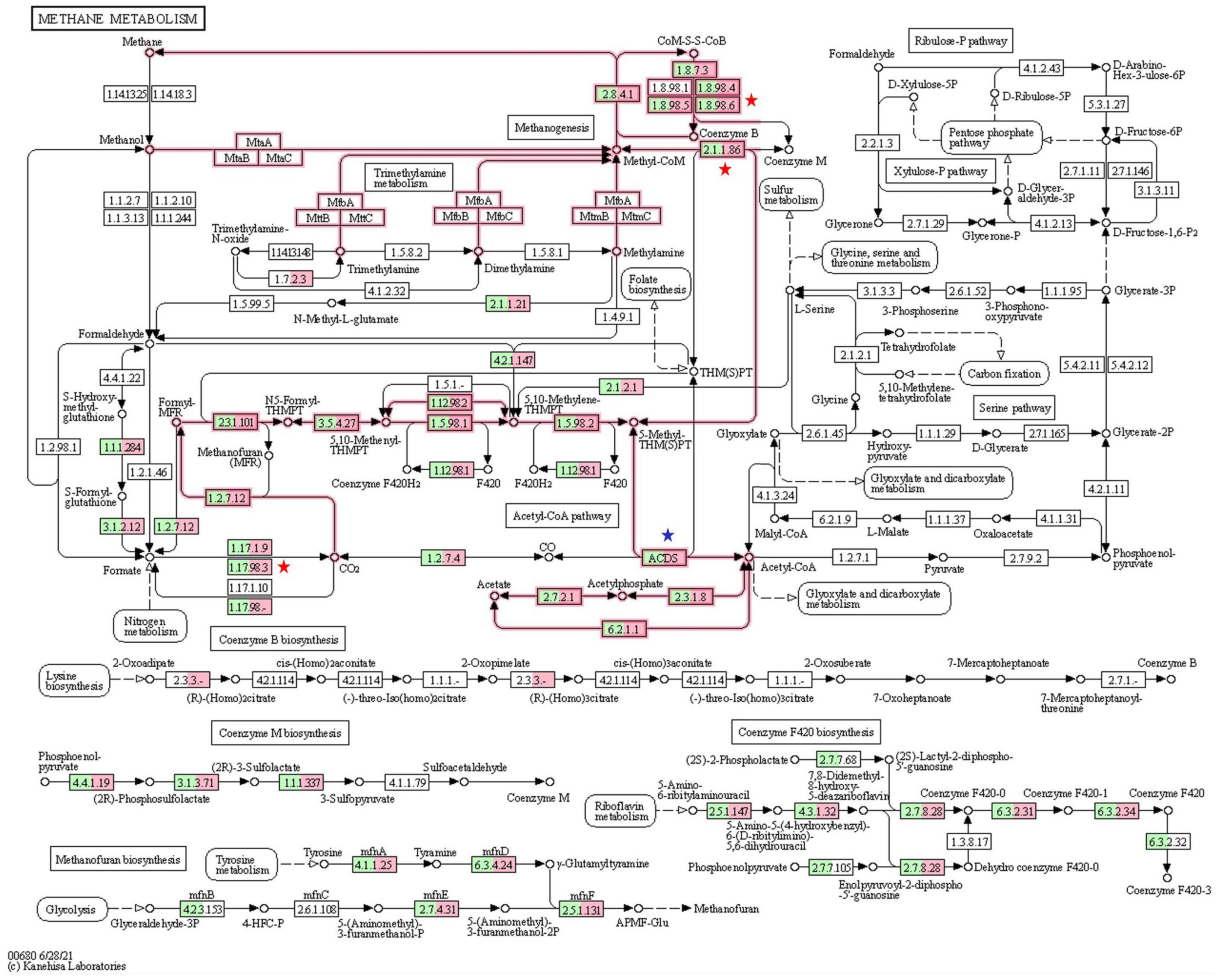
KEGG analysis for methanogenesis pathways. The numbers in the boxes represent the EC numbers of enzymes involved in methanogenesis pathways. The green color inside the box represents relative genes from the CON group microbiome, while the box in pink refers to the relative genes from the HD treatment group. At several steps in the pathway, genes related to methane metabolism has been significantly reduced in the HD treatment group. KO numbers and gene annotation of the enzymes are shown in Supplementary File.

KEGG analysis revealed that all three methanogenesis pathways (hydrogenotrophic, methylotrophic, and aceticlastic) were active in both CON and HD treatment groups (Figure 6). A total of 36 KO related to methanogenesis were identified in this study (Supplementary File 1). The EC 1.8.98.6 (formate dehydrogenase) assigned to a total of 6 KO was less abundant in the HD treatment group. Out of six KO, the abundance of K22516 (*p* = 0.029) and K00125 (*p* = 0.044), representing alpha and beta units respectively, were significantly lower in the anti-methanogenic supplemented group. However, the abundance of K03388, K03389, K03390, and K14127 was not different between the groups. The KEGG enzyme 2.1.1.86 (tetrahydromethanopterin S-methyltransferase), composed of eight different subunits, was also significantly different between the two groups. More specifically, the abundance of K00581 (subunit E, *p* = 0.027) and K00584 (subunit H, *p* = 0.037) was lower in the HD treatment group. The KEGG analysis did not find any difference in the number of other subunits (A-D, F-G).

K00125 (p = 0.044) and K22516 (p = 0.029) assigned to the KEGG enzyme 1.17.98.3 (formate dehydrogenase) were significantly lower in the HD treatment group. Results from the study indicated that K22516 and K00125 were assigned to two KEGG enzymes, EC1.8.98.6 and EC 1.17.98.3. On the contrary, the supplementation of anti-methanogenic product in the HD treatment group increased the abundance of ACDS (acetyl CoA decarbonylase, EC 2.3.1.169). A total of three KO (K00193, K00194, and K00197) enzymes were assigned to the ACDS and, among these, only K00197 was significantly different (*p* = 0.042) between the two groups.

## Discussion

A significant reduction (~22%) in the daily enteric methane emission in sheep demonstrated that the anti-methanogenic supplement *Harit Dhara* has tremendous potential for the mitigation of enteric methane up to the desirable limit where ruminal fermentation is not compromised. Ruminal microorganisms reside in the ecosystem in a syntrophic manner to perform a specific functional task. The relationship between ruminal protozoa and methanogens is an excellent illustration of syntrophy. Methane mitigation by the supplementation of tanniferous sources is achieved in both indirect and direct manners (Bhatta et al., 2009; Gemeda and Hassen, 2015). The indirect inhibition is mediated by halting the interspecies H_2_ transfer because of the decrease in rumen protozoa (Malik et al., 2017a,b; Baruah et al., 2019). A significant reduction in the numbers of protozoa in the HD treatment group, associated with a decrease in methane emissions, revealed that protozoa were one of the most significant factors contributing to rumen methanogenesis. Previous studies on sheep (Malik et al., 2017a,b; Baruah et al., 2019; Poornachandra et al., 2019) support our findings that a considerable reduction in enteric methane emissions can be attributed to the reduction in ruminal protozoa due to tanniferous phyto-sources. Further, the comparison of tannin-driven partial defaunation in this paper with the previous studies (Qin et al., 2012; Guyader et al., 2014; Nguyen et al., 2016) reported partial defaunation with other agents other than tanniferous sources also revealed a similar reduction in the enteric methane emission.

Protozoa are abundant but unwanted inhabitants of the rumen (Morgavi et al., 2012). The digestion of fiber could be negatively impacted by the defaunation (Ushida et al., 1990), as they appear to have better carboxymethylcellulase activity than bacteria (Coleman, 1985). On the other hand, some studies revealed no difference (Jouany, 1996; Han et al., 1999), or even increased fibrolytic activity due to the defaunation (Soetanto et al., 1985; Romulo et al., 1986). Despite their significant representation in the rumen microbiota, the role of protozoa in fiber digestibility is still controversial and debatable (Williams and Coleman, 1992). The increased propionate production in the HD treatment group can be due to the partial defaunation that promotes the development of succinate-producing bacteria (Kurihara et al., 1978; Li et al., 2020).Major succinate-producing bacteria in the rumen are *Ruminicoccus flavefaciens*, *Bacteroides succinogenes* and *Selenomonas ruminantium* (Scheifinger and Wolin, 1973). The relative abundance comparison of the major succinate producing bacteria in our study revealed the marginally higher distribution of *Ruminicoccus* (CON 2.9 vs. HD 3.3%), and *Selenomonas* (CON 0.43 vs. 0.47%) at the genus level (Supplementary File 1). The anti-methanogenic supplement offered to the HD treatment group lowered protein breakdown, as indicated by the significantly lower rumen ammonia concentration. It is a well-known fact that when tannin sources are added, nitrogen breakdown moves from the rumen to the intestine (Orlandi et al., 2015). Additionally, defaunation may be one of the causes of the reduced ammonia concentration in the rumen (Newbold et al., 2015).

The extent of volatile fatty acids production is primarily dependent on organic fermentation (Dijkstra, 1994) and remains the same until the diet composition in terms of the proportion of structural and soluble carbohydrates does not vary. The basal diet in the CON and HD treatment groups was similar in respect of ingredients and their proportions, and therefore the organic matter intake and digestibility in the present study also did not vary between the two groups. The similar diet composition, intake and digestibility, particularly of organic matter, can be accountable for the similar volatile fatty acids production. However, the sheep in the HD treatment group additionally received the anti-methanogenic supplement, primarily consisting of condensed tannins. The adverse impact of the tannins on the fermentation has been reported, particularly when supplemented at such high levels (Kumar and Vaithiyanathan, 1990). In a recent study, Guerreiro et al. (2021) reported that condensed tannin adversely impacted volatile fatty acid production; however, the adverse impact was more pronounced at higher doses (75–100 g/kg DM). The concentration of tannins (10.4 g/kg DM) in the HD treatment group study was not adequate to induce an adverse impact on the fermentation of organic matter and the subsequent volatile fatty acids production. These findings are consistent with the findings of Bhatta et al. (2015), who reported that tannin supplementation has no effect on volatile fatty acid production at low levels.

The similar blood profile between CON and HD treatment groups shows that ingesting tannin-rich anti-methanogenic supplement at the appropriate level had no effect on the blood’s enzymes, proteins, or minerals (Supplementary Table 3). Our results are supported by earlier research (McSweeney et al., 1988; Makkar, 2003), which concluded that the condensed tannin is not absorbed into the bloodstream and can damage an organ if it enters the bloodstream in the event of intestinal damage. On the other hand, Zhao et al. (2019) reported that Hu sheep fed on maize straw and a concentrate-base diet supplemented with 0.1% tannin had a significantly higher level of globulin and alkaline phosphatase as compared to control.

Other ways in which tanniferous phyto-sources limit intestinal methanogenesis include a reduction in fiber digestion and ruminal fermentation (McSweeney et al., 2001; Tavendale et al., 2005; Mueller-Harvey, 2006). However, similar feed intake, digestibility, and volatile fatty acid concentrations across the CON and HD treatment groups did not support that the decrease in methane in the present study was due to reduced fiber digestion. The reduction in methane emissions resulting from poor fiber digestion is dependent on the concentration (Chiquettei et al., 1988; Tan et al., 2011), molecular size (McLeod, 1974), and type of tannins (Getachew et al., 2008; Bhatta et al., 2009). Therefore, the optimal concentrations of tannins in the diet led to a reduction in methane emissions without affecting fiber digestion or ruminal fermentation (Carulla et al., 2005; Tavendale et al., 2005). The concentration of tannins in the HD treatment group at the given intake was 10.4 g/kg DM, which is significantly below the concentration of 20 g/kg DM (Waghorn et al., 1987), at which tannins exert the most detrimental effect on fermentation. The reduction in methane output without impairing intake and fermentation in our investigation corroborated previous findings in sheep supplemented with a nearly same concentration of tannins (Malik et al., 2017a,b; Baruah et al., 2019; Poornachandra et al., 2019).

Further, the comparable core bacterial communities, such as *Bacteroidetes* and *Firmicutes* between the groups also demonstrated that the anti-methanogenic supplement did not affect the fiber digestibility in the sheep rumen. The composition of the bacterial metagenome in this study was consistent with previous reports (Khafipour et al., 2009; Jami and Mizrahi, 2012; McLoughlin et al., 2020; Malik et al., 2022). However, the anti-methanogenic supplement in the HD treatment group specifically decreased the abundance of *Proteobacteria*, which can be ascribed to the tannins antibacterial properties. Our findings are in good agreement with the previous studies (Taguri et al., 2004; Yao et al., 2006; Min et al., 2007), which reported the adverse impact of tannins on *Proteobacteria*. Tannins act mainly on the microbial cell membrane (Liu et al., 2013). However, this action is microbe species-specific and closely linked to the structure (Huang et al., 2018). The antibacterial effect of condensed tannins against *Proteobacteria*, such as *Escherichia* and *Shigella*, is well documented (Funatogawa et al., 2004; Banso and Adeyemo, 2007; Liu et al., 2013).

Conversely, despite the lower abundances of *Lentisphaerae* (0.47–0.67%), we observed significant differences in abundances between the two groups. The low abundance of *Lentisphaerae* in the rumen of sheep is consistent with previous findings (Myer et al., 2015; Lourenco et al., 2020). It has been reported that bacteria belonging to the phylum *Lentisphaerae* are linked to feed efficiency (Mao et al., 2013; Guan et al., 2017). In a study, Ahmad et al. (2020) demonstrated a linear relationship between the abundance of *Lentisphaerae* and the energy levels in yak. The greater production of volatile fatty acids, an indicator of energy, also revealed a greater *Lentisphaerae* abundance in the HD treatment group. Nevertheless, further confirmation is necessary to establish the mechanism and role of *Lentisphaerae* in the overall feed conversion efficiency.

The supplementation of tanniferous sources directly inhibited methanogens, resulting in a substantial decrease in rumen methanogenesis. A few well-described mechanisms of tannins action on rumen archaea that result in methane reduction include compositional change in cell membranes, enzymatic inhibition, and lack of substrate and metallic ions (McSweeney et al., 2001). Tannin exerts a detrimental effect on methanogens by tanning proteins at accessible sites inside or on methanogens (Field and Lettinga, 1987; McSweeney et al., 2001). The tanniferous anti-methanogenic supplement significantly decreased the abundance of ruminal archaea such as *Methanocaldococcus*, *Methanococcoides*, *Methanocella*, and *Methanoregula* in the HD treatment group as compared to that of CON (3.3 vs. 4.4%). Archaea from the domain *Euryarchaeota* constitute 3%–5% of the rumen microbiota (Yanagita et al., 2000; Henderson et al., 2015; Malik et al., 2021). The dominance of *Methanobacteriales* as dominant archaea in the sheep rumen is in good agreement with the previous reports (Malik et al., 2022; Thirumalaisamy et al., 2022a). Similarly, our results on the dominance and abundance of *Methanobrevibacter* is in line with our previous research (Baruah et al., 2018; Malik et al., 2022; Thirumalaisamy et al., 2022a, 2022b).

A significant decrease in the abundances of *Methanocaldococcus*, *Methanococcoides*, *Methanocella*, and *Methanoregula* in the HD treatment group warrants further research to determine how tanniferous material affects their abundance. A recent report (Kibegwa et al., 2022) revealed that the distribution of *Methanococcus* in the rumen is influenced by the diet composition and its distribution tends to be greater on high roughage diets than on concentrate diets. However, the contribution of *Methanocaldococcus*, *Methanococcoides*, *Methanocella*, and *Methanoregula* to ruminal methanogenesis must be determined before concluding that their decreased abundance contributed to the lowering of methane emissions.

The *Crenarchaeota*-affiliated methanogens identified in this study have been reported previously in the rumen (Janssen and Kirs, 2008; Zhao et al., 2020; Malik et al., 2021). However, information regarding their participation in and contribution to rumen methanogenesis is scanty. *Korarchaeaota* is the third and least-characterized archaeal phylum (Barns et al., 1994, 1996). This phylum is typically found in terrestrial hot springs (Spear et al., 2005; Takai and Sako, 2006; Elkins et al., 2008) and marine hydrothermal vents (Marteinsson et al., 2001; Rogers et al., 2005). *Korarchaeaotes* are effective peptide degraders (Elkins et al., 2008), and their inability to synthesize cofactors, vitamins, and purines renders them either a symbiotic microbe or an effective scavenger (Nealson, 2008). This is, to the best of our knowledge, the first study to report the presence of *Korarchaeaota* in the rumen.

*Thaumarchaeota* was previously classified as a subgroup of *Crenarchaeota*; however, *Cenarchaeum symbiosum* genome sequencing in 2008 resulted in its classification as a separate archaeal phylum. The analysis of conserved signature indels (CSIs) and signature proteins provided insights into the distinction between *Thaumarchaeota* and *Crenarchaeota* (Gupta and Shami, 2011). *Thaumarchaeotes* are the dominant ammonia-oxidizing archaea in soil systems (Schleper and Nicol, 2010) and in the deep sea (Zhong et al., 2020). In a previous study (Deusch et al., 2017), based on tryptic peptide analysis (meta-proteome), the *Thaumarchaeota* phylum was identified in the rumen of Jersey cows fed corn or grass silage and/or grass hay in a rotational manner. The occurrence of *Thaumarchaeota* in our study at an extremely low frequency is in congruence with a previous report (Deusch et al., 2017). On the contrary, Wang et al. (2016) reported that *Thaumarchaeota* was one of the dominant phyla and constituted 15% of the total archaea community in the goat rumen. They conclude that *Thaumarchaeotes* participate in the oxidation of ammonia to nitrite and, due to their acid tolerance ability, thrive better on a high-concentrate diet. Nevertheless, this could not be substantiated by comparing the findings of Wang et al. (2016) with those of Deusch et al. (2017), despite the fact that the latter study contained more protein than the former. Due to the limited number of studies, the correlation between *Thaumarchaeota* distribution frequency and diet cannot be confirmed and requires further investigation.

The abundance of KEGG orthologs K00581 and K00582 representing tetrahydromethanopterin methyltransferase (EC 2.1.1.86) was significantly lower in the HD treatment group. This enzyme is accountable for the methyl transfer to coenzyme M (Gottschalk and Thauer, 2001). More specifically, the lower abundance of E and F subunits of the enzyme might lead to a sodium ion translocation-driven reduction in methanogenesis. Similarly, the KEGG orthologues K22516 and K00125, accountable for the activity of formate dehydrogenase (EC 1.8.98.6 & EC 1.17.98.3), were less abundant in the HD treatment group. This enzyme facilitates electron transfer to the HdrA subunit (Thauer, 2012), which might have reduced the reduction of ferredoxin and thereby methanogenesis. In contrast, the higher abundance of K00197, accountable for the acetyl-CoA decarbonylase/synthase complex (EC 2.3.1.169) in the HD treatment group, might have increased the transfers of methyl group (Li et al., 2013). However, in this study, we have not checked the activity of the above enzymes *per se* and are not sure if the lower abundance was transcribed.

KEGG analysis results revealed that the activity of the enzymes such as formate dehydrogenase and tetrahydromethanopterin methyltransferase involved in the formation of methane from CO_2_/formate through a hydrogenotrophic pathway was comparatively less in the HD treatment group. This likely contributed to a reduction in methanogenesis as a whole. *Methanocella* and *Methanoregula* are hydrogenotrophic methanogens that utilize H_2_/CO_2_ for methane production (Sakai et al., 2008; Yashiro et al., 2011); consequently, the partial inhibition of hydrogenotrophic methanogenesis in the present study can be attributed to overall less methanogens population as revealed by qPCR and the low abundances of the above two methanogens in the HD treatment group. Similarly, the lower distribution of obligatory methylotrophic *Methanococcoides* (Franzmann et al., 1992) in the HD treatment group should account for the reduced methane emission in sheep fed on an anti-methanogenic supplement-based diet. Due to the absence of pure cultures and the fact that the total abundance of these methanogens in the rumen is 5%, it is extremely difficult to determine the contribution of a particular genus to the overall rumen methanogenesis. To the greatest extent possible, ruminal archaea must be cultured and the roles of *Methanococcoides* and hydrogenotrophic genera (*Methanocella* and *Methanoregula*) in rumen methanogenesis must be investigated.

Since this product was formulated with readily available, inexpensive agricultural phyto-sources, has little food-feed competition and could therefore be one of the promising products for the small and marginal farmers to reduce the negative environmental consequences of livestock production and productivity enhancement.

## Conclusion

From the study, it can be inferred that the anti-methanogenic supplement (HD treatment) at the 5% level in the straw and concentrate-based diet led to a significant decrease (~22%) in the enteric methane emission without compromising the intake and rumen fermentation. The supplement has the ability to significantly reduce ruminal protozoa and concurrently lower methane emissions. The comparable makeup of bacterial and archaeal metagenomes between groups revealed that the supplement had no effect on the core microbiome and the modest alterations in prokaryotic communities were due to minor species. Despite the vulnerability of minor methanogens in the archaeal community, the anti-methanogenic supplement greatly decreased the activity of key enzymes such as formate dehydrogenase and tetrahydromethanopterin S-methyltransferase involved in rumen methanogenesis. The culturing of minor rumen methanogens, such as *Methanocaldococcus*, *Methanococcoides*, *Methanocella*, and *Methanoregula*, should be taken up as a priority, and we should look into the impact of these archaea on the overall rumen methanogenesis. This product is formulated with readily available, inexpensive agricultural waste phyto-sources, has little food-feed competition, and could be one of the promising tools for small and marginal farmers to reduce the negative environmental impact of livestock production while improving the productivity.

## Data availability statement

The datasets presented in this study can be found in online repositories. The names of the repository/repositories and accession number(s) can be found at: https://www.ncbi.nlm.nih.gov/bioproject/, PRJNA885963.

## Ethics statement

After obtaining prior approval (NIANP/IAEC/1/2019) from the Institute Animal Ethics Committee (IAEC), a study in sheep was conducted in strict accordance with the IAEC’s procedures for animal care and sample collection.

## Author contributions

PM, RB, and AK conceived and designed the study. PM, ST, and AM performed the *in vitro* and *in vivo* studies and executed the chemical composition and ruminal fluid analysis. AK and ST performed the molecular and bioinformatics analysis as well as visualization of the data. RB and HR acquired the required funding from the ICAR and ILRI. PM and RB wrote the draft and final version of the manuscript. All authors contributed to the article and approved the submitted version.

## Funding

The authors are grateful to the Indian Council of Agricultural Research, New Delhi, for providing financial support for the project entitled “Estimation of methane emissions under different feeding systems and development of mitigation strategies” under which the anti-methanogenic supplement was developed and preliminary research was conducted. The authors are also grateful to the International Livestock Research Institute (ILRI), Nairobi, for funding the animal studies for the evaluation of the product under the project “Methane emissions and its mitigation.”

## Conflict of interest

The authors declare that the research was conducted in the absence of any commercial or financial relationships that could be construed as a potential conflict of interest.

## Publisher’s note

All claims expressed in this article are solely those of the authors and do not necessarily represent those of their affiliated organizations, or those of the publisher, the editors and the reviewers. Any product that may be evaluated in this article, or claim that may be made by its manufacturer, is not guaranteed or endorsed by the publisher.

## Supplementary material

SUPPLEMENTARY FIGURE 1 Effect of anti-methanogenic supplement on the total methanogens (log2).

SUPPLEMENTARY FILE 1 Bacterial abundance, Archaeal abundance, KO Abundance, Statistical comparison results of Bacterial, Archaeal genera and KOs.
