# Supplementary figures and images for: Effect of an anti-methanogenic supplement on enteric methane emission, fermentation, and whole rumen metagenome in sheep
Source: Front Microbiol. 2022 Nov 21;13:1048288. doi: 10.3389/fmicb.2022.1048288 (PMC9719938; doi:10.3389/fmicb.2022.1048288)

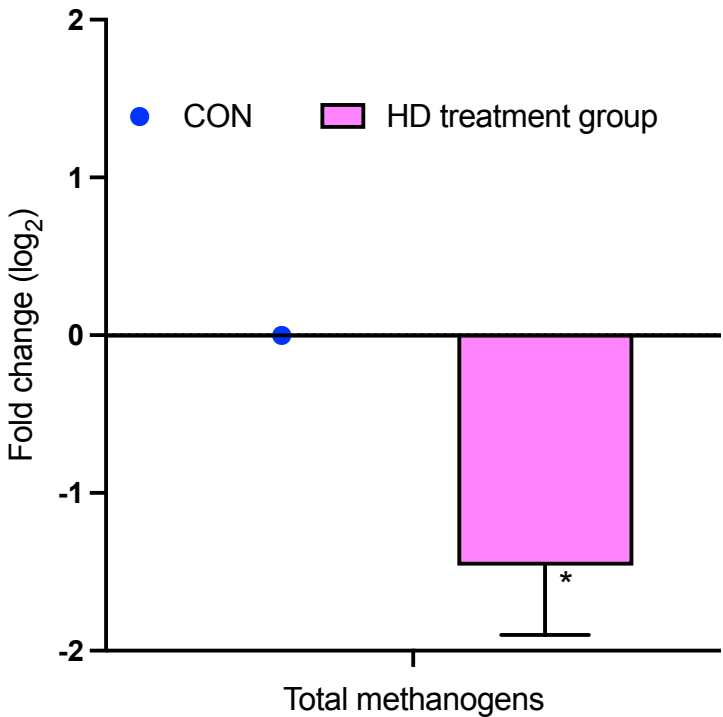

Supplement: SUPPLEMENTARY FIGURE 1 — Effect of anti-methanogenic supplement on the total methanogens (log2). [file Data_Sheet_2.PDF]
