# Supplementary material for: Effect of an anti-methanogenic supplement on enteric methane emission, fermentation, and whole rumen metagenome in sheep
Source: Front Microbiol. 2022 Nov 21;13:1048288. doi: 10.3389/fmicb.2022.1048288 (PMC9719938; doi:10.3389/fmicb.2022.1048288)
Supplement: Supplementary file 3 [file Table_1.DOCX]

Supplementary Table 1: Chemical composition of various diets for in vitro study

| **Treatment** | **Constituents (g/kg DM)** | | | | |
| --- | --- | --- | --- | --- | --- |
|  | OM | CP (Nx6.25) | NDF | ADF | Ash |
| CON | 920 | 122 | 555 | 314 | 80 |
| HD_2_ | 919 | 116 | 573 | 323 | 81 |
| HD_5_ | 911 | 120 | 603 | 337 | 89 |
| HD_8_ | 908 | 121 | 636 | 350 | 92 |

OM- organic matter, CP- crude protein, NDF- neutral detergent fibre, ADF- acid detergent fibre; CON- control (without anti-methanogenic supplement), HD_2_, HD_5_ and HD_8_ were the treatments where anti-methanogenic supplement was added over and above to the control diet at the level of 2, 5 and 8%, respectively. The OM, CP, NDF and ADF content of the anti-methanogenic supplement was 943, 68.8, 587 and 373 g/kg, respectively.
