# Supplementary material for: Effect of an anti-methanogenic supplement on enteric methane emission, fermentation, and whole rumen metagenome in sheep
Source: Front Microbiol. 2022 Nov 21;13:1048288. doi: 10.3389/fmicb.2022.1048288 (PMC9719938; doi:10.3389/fmicb.2022.1048288)
Supplement: Supplementary file 4 [file Table_2.DOCX]

Supplementary Table 2: Chemical composition of the basal diet*

| **Attributes** | **Proportion** | **Composition (g/kg)** | | | | | |
| --- | --- | --- | --- | --- | --- | --- | --- |
|  |  | **DM** | **OM** | **Ash** | **CP** | **NDF** | **ADF** |
| Finger millet straw | 50 | 890 | 910 | 90 | 40.2 | 714 | 494 |
| Concentrate | 50 | 915 | 945 | 55 | 193 | 411 | 122 |
| Basal diet | 100 | 901 | 927 | 73 | 124 | 556 | 314 |
| **Concentrate ingredients** | | | | | | | |
| Maize grain | 32 | 906 | 979 | 21 | 88 | 494 | 91.3 |
| Groundnut cake | 12 | 924 | 944 | 56 | 412 | 260 | 178 |
| Soybean | 13 | 922 | 912 | 88 | 449 | 319 | 140 |
| Wheat bran | 40 | 927 | 956 | 44 | 131 | 431 | 132 |
| Mineral mixture | 2 | - | - | - | - | - | - |
| Salt | 1 | - | - | - | - | - | - |

* Anti-methanogenic supplement *Harit Dhara* was added to the basal diet @ 5% of intake in test group (HD).
