# Supplementary material for: Effect of an anti-methanogenic supplement on enteric methane emission, fermentation, and whole rumen metagenome in sheep
Source: Front Microbiol. 2022 Nov 21;13:1048288. doi: 10.3389/fmicb.2022.1048288 (PMC9719938; doi:10.3389/fmicb.2022.1048288)
Supplement: Supplementary file 5 [file Table_3.DOCX]

Supplementary Table 3: Effect of Harit Dhara supplementation on blood profile

| **Attributes** | **CON** | **HD** | **SEM** | **P** |
| --- | --- | --- | --- | --- |
| **Enzyme** | | | | |
| Alkaline Phosphatase (U/l) | 145 | 224 | 24.06 | 0.104 |
| Alanine aminotransferase (U/l) | 98.83 | 104.5 | 5.78 | 0.647 |
| γ gultamyl transferase (U/l) | 44.83 | 43.83 | 2.03 | 0.819 |
| Creatine kinase (U/l) | 239.0 | 180.0 | 17.76 | 0.101 |
| **Protein** | | | | |
| Albumin (g/l) | 46.16 | 38.40 | 0.570 | 0.202 |
| Globulin (g/l) | 33.50 | 31.83 | 1.76 | 0.659 |
| Total protein (g/l) | 79.83 | 79.33 | 1.76 | 0.895 |
| Blood urea nitrogen (mg/dl) | 4.75 | 5.17 | 0.158 | 0.200 |
| **Mineral** | | | | |
| Ca (mmol/l) | 2.40 | 2.36 | 0,04 | 0.678 |
| P (mmol/l) | 2.36 | 2.59 | 0.143 | 0.443 |
| Mg (mmol/l) | 1.03 | 0.96 | 0.02 | 0.206 |

CON- control group (no *Harit Dhara* supplementation); HD- test group (*Harit Dhara* supplementation @ 5% basal diet); U/l- unit per litre; g/l- gram per litre; mM/l- milli mole per litre; Ca- calcium; P- phosphorus; Mg- magnesium; SEM- standard error of mean
